# Effects of green space on physical activity and body weight status among Chinese adults: a systematic review

**DOI:** 10.3389/fpubh.2023.1198439

**Published:** 2023-07-20

**Authors:** Yiling Song, Haoxuan Li, Hongjun Yu

**Affiliations:** Department of Physical Education, Tsinghua University, Beijing, China

**Keywords:** green space, physical activity, body weight, China, systematic review

## Abstract

**Background:**

Green space may provide many benefits to residents’ health behaviors and body weight status, but the evidence is still relatively scattered among Chinese adults. The purpose of this study was to review the scientific evidence on the effects of green space on physical activity (PA) and body weight status among Chinese adults.

**Methods:**

A keyword and reference search was conducted in Pubmed, Web of Science, MEDLINE, and PsycINFO. Studies examining the associations between green space and PA, body mass index (BMI) among Chinese adults were included. The quality of the included literature was evaluated using the National Institutes of Health’s Observational Cohort and Cross-Sectional Study Quality Assessment Tool.

**Results:**

A total of 31 studies were included that met the inclusion criteria, including 25 studies with a cross-sectional design, 3 studies with a longitudinal design, and 3 studies with an experimental design. Street-level green view index and green space accessibility were found to be positively associated with PA, but negatively associated with BMI. In most studies, there was a correlation between green space ratio in local areas and BMI. In addition, green space interventions were effective in increasing PA and decreasing BMI among Chinese adults. In contrast, further evidence is needed to support the association between the design characteristics of green space and PA and BMI.

**Conclusion:**

Preliminary evidence suggests that green space has a positive effect on PA and BMI among Chinese adults. However, there are contradictory findings, and future studies adopting longitudinal and quasi-experimental studies are needed to further explore the causal relationship between green space and PA and BMI to provide a relevant theoretical basis for policymakers.

## 1. Introduction

There is evidence that physical inactivity is associated with an increased risk of chronic disease, leading to obesity, diabetes, hypertension, heart disease, and other chronic diseases (1). Conversely, sufficient physical activity (PA) and regular PA can prevent and reduce the risk of chronic disease and contribute to a higher quality of life. PA is defined as physical movement in which muscle contraction causes energy expenditure (2). The energy expenditure of a person while sitting still is approximately 1 metabolic equivalent (MET). Moderate physical activity (MPA) usually has a range of ≥3METs and < 6METs (e.g., brisk walking, dancing, etc.); moderate to vigorous physical activity (MVPA) is defined as requiring ≥3METs of energy expenditure (e.g., brisk running, fast cycling, etc.) (3). The World Health Organization (WHO) reports that physical inactivity is the fourth leading risk factor for global mortality, further contributing to the prevalence of non-communicable diseases (4). In addition, previous studies show that nearly 2 billion adults worldwide are considered overweight, more than half of whom are classified as obese (5, 6). Obesity is associated with the development of many diseases such as atherosclerosis, type 2 diabetes, and dyslipidemia, and has become a global epidemic and public health crisis (7, 8). Previous studies have reported that chronic diseases caused by physical inactivity and obesity not only have a serious negative impact on the physical and mental health of individuals but also create a heavy burden on social development (9–12). Therefore, WHO and professional health agencies expect to develop active and effective intervention policies to help people improve their PA and adopt healthy weight.

Available evidence suggests that environmental interventions can help people achieve a balanced lifestyle in their daily lives (13–15). In terms of PA, it can be done in an indoor environment or an outdoor environment. However, existing research has found that PA in an outdoor environment has greater benefits for human physical and mental health than in an indoor environment (16). Green spaces are an important part of the outdoor environment and include greenways, parks, gardens, street greenery and land covered with grass, shrubs, trees, or other vegetation (17). Green spaces are associated with PA among residents (18, 19). Several studies have shown that green spaces have a positive impact on people’s physical and mental health, and can promote PA, reduce sedentary behavior, and lower overweight and obesity (20–23). For example, one study showed that community residents tend to spend more time outdoors on weekdays and weekends, and also do more MVPA and vigorous activity, if they live in neighborhoods with more green spaces (24). Another study of Norwegian adults showed that living in a green community had a positive impact on increased PA among adults (25). According to a study of Danish adults, proximity to green space was associated with PA and obesity, and maintaining one’s physical fitness was a key factor in choosing to visit green spaces (26). A study from American showed that BMI in adults was associated with forested land areas and proximity to public recreational areas (27). However, the current findings are inconsistent, such as a study from Portugal that did not find an association between the amount of urban green space, green exposure, and the prevalence of overweight and obesity in the Portuguese adolescent population (28). The effect of green space on PA and the obesity of residents still deserves further research.

China is rapidly urbanizing, modernizing, and growing economically. However, physical inactivity, sedentary behavior, being overweight, and obesity are prevalent among Chinese adults in the urbanizing and modernizing Chinese society (29, 30). From 1991 to 2006, PA levels among Chinese adults declined at an unprecedented rate, with average weekly PA declining by 32%, and the largest percentage decline in PA in China compared to the United States, United Kingdom, India, and Brazil (31). In addition, since the 1990s, China has seen numerous significant social, economic, and lifestyle changes, which have coincided with the rapidly increasing prevalence of obesity. Over half of the adults and a fifth of children are overweight or obese, making China the nation with the highest proportion of such people globally (32). Consequently, the problem of physical inactivity and obesity among Chinese adults needs to be addressed.

Attempts to promote resident health behaviors through environmental interventions are receiving increasing attention, and green spaces may play an important role in environmental interventions. Despite the aforementioned work, two major gaps in the previous studies remain. First, research on green space and resident PA and obesity have focused on developed countries, with fewer studies targeting developing countries. For example, an American study showed that tree cover on streets was associated with active traffic behavior among residents and that a 10% increase in tree cover on streets was associated with a 19%–41% increase in the odds of active traffic (33). In addition, a study from England showed that people living in the greenest areas of England were more likely to achieve the recommended level of PA (34). The characteristics and quality of green space may vary from country to country due to differences in urbanization level, economic level, spatial scale, and geographic location, which may have different effects on the health behaviors of residents. For these reasons, research findings and recommendations for developed countries are not necessarily applicable to developing countries such as China. Therefore, in order to help establish a global framework for the use of green space, developing countries also need to actively contribute to scientific research.

Second, China is experiencing rapid urbanization and is facing serious physical inactivity and overweight/obesity problems, which according to existing studies are found to be regulated and controlled by environmental interventions. However, the results of current studies on the effects of green space on PA and the body weight status of Chinese residents are varied. The inconsistent results of previous studies challenge relevant policy makers and urban planners to develop targeted environmental intervention programs to improve the physical activity levels of residents. Therefore, a systematic review of existing studies is needed to better summarize the findings and limitations of existing studies and to make recommendations for future environmental interventions. In addition, the effects of green space on PA and the body weight status of residents may also vary by population, and no systematic review has been conducted specifically for Chinese adults.

Therefore, the purpose of this study was to systematically review the literature on the effects of green space on PA and body weight status in Chinese adults, to review the findings of existing studies, and to suggest future research trends. It also aimed to identify the research gaps in this field. The results of this review can provide a targeted theoretical basis for policymakers and stakeholders of environmental interventions.

## 2. Methods

This systematic review followed the recommendations provided by the Preferred Reporting Items for Systematic Reviews and Meta-Analysis guidelines (35).

### 2.1. Literature search

A comprehensive literature search of four electronic literature databases (Pubmed, Web of Science, MEDLINE, and PsycINFO) was conducted simultaneously. The search strategy was based on a combination of subject terms and free terms. It was determined after repeated pre-checks and supplemented with manual searches, retroactively including references to the literature when necessary. The search dates range from 2000 to February 21, 2023. Take PubMed as an example, and the specific search strategy is shown in Table 1.

**Table 1:** Pubmed search policy.

Serial no.	Search contents
#1	green space* OR greenland OR greenbelt OR greenness OR greenspace OR park OR parks OR open space* OR nature OR public space* OR garden* OR street tree*
#2	physical activ* OR exercis* OR walk* OR cycl* OR outdoor activity OR leisure OR health behav* OR motor activity OR motor activities OR overweight or obesity or obese or adiposity or body fat OR body mass index OR BMI OR body weight
#3	China or Chinese
#4	#1 and #2 and #3

### 2.2. Inclusion and exclusion criteria

The inclusion criteria were as follows: (1) study subjects—Chinese adults aged ≥18 years; (2) study designs—observational studies (e.g., cross-sectional studies or longitudinal studies), experimental studies, and qualitative studies; (3) exposures—different types and metrics of green space (e.g., parks, green street, or vegetation areas); (4) outcomes—PA (e.g., leisure PA, MPA, MVPA) and/ or body weight status (e.g., BMI); (5) language—Papers published in English; (6) article type—peer-reviewed publications; (7) country—China.

The exclusion criteria were as follows: (1) studies that did not include PA or body weight status as an outcome; (2) repeatedly published literature with poor quality assessment; (3) studies included Chinese subjects who resided outside of the Chinese Mainland, Hong Kong, or Macao; (4) case reports, review literature, conference papers, and dissertations; (5) non-English papers.

### 2.3. Study selection

The two researchers screened the literature independently according to the inclusion and exclusion criteria. First, the papers were initially screened by reading the title and abstract. Second, the full text was downloaded after obtaining the eligible literature, and full-text screening was performed. Finally, the two researchers compared the independently screened literature. For the literature with inconsistent screening results, the decision to include or not was to be made by a joint discussion with the third researcher.

### 2.4. Data extraction and synthesis

Information related to the included literature was extracted independently by two researchers using a standardized form. The extracted information was as follows: (1) basic information: first author, year of publication, and region; (2) basic characteristics of the participants: age, sample size, the proportion of females, sample characteristics; (3) statistical analysis: statistical model, nonresponse rate; (4) others: geographical coverage, type of green space measure, the detailed measure of green space, type of PA measure, detailed measure of PA, estimated effects of green space on PA, type of body weight status measure, the detailed measure of body weight status, estimated effects of green space on body weight status, and key findings. Because heterogeneous exposure and outcome metrics hindered Meta-analysis, we provided a narrative summary of common themes and findings from the included studies.

### 2.5. Study quality assessment

Each included study’s quality was evaluated using the National Institutes of Health’s Quality Assessment Tool for Observational Cohort and Cross-Sectional Studies (36). And each included study has 14 questions for the quality evaluation. For each question, “yes” responses received a score of one, while “no” responses received a score of zero. By computing the results for each criterion, a study’s overall quality score is determined. The strength of the scientific evidence was measured using study quality assessment, however, the inclusion of research was not taken into consideration.

## 3. Results

### 3.1. Search results

**Figure 1:**
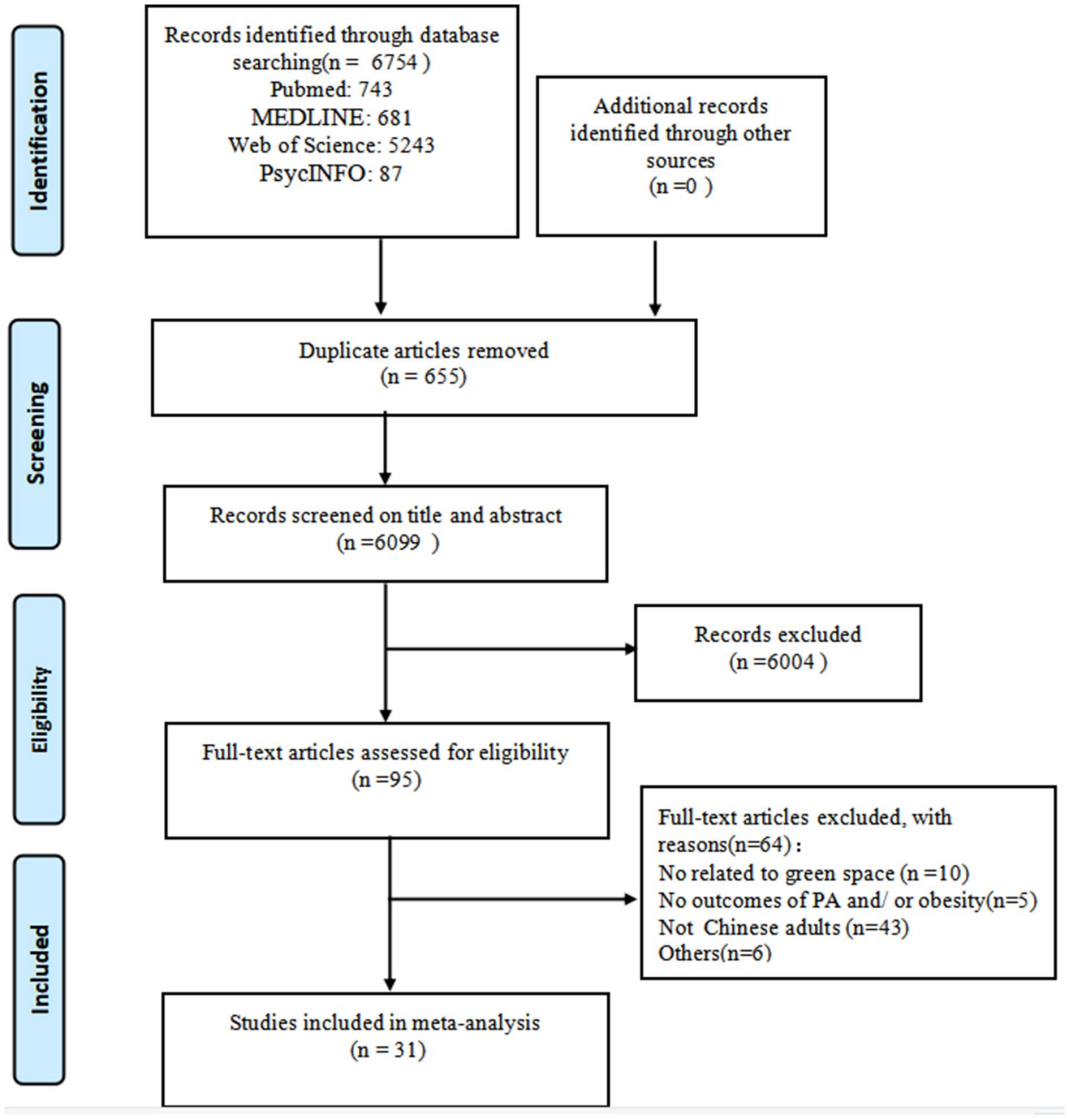
Flow diagram of study selection.

A total of 6,754 articles were retrieved from the database, and after removing duplicates, 6,099 articles were obtained. In the literature screening process, firstly, a total of 6,004 articles that did not match the research topic were removed by reading the titles and abstracts. Second, the remaining 95 articles were read in full text, among which 10 articles had environmental exposure indicators unrelated to green space, 5 articles had outcome variables that did not include physical activity and/weight status, 3 articles had study subjects that were not Chinese adults, and 6 other articles were excluded due to poor quality of literature and conference papers, and a total of 31 articles were finally included. Figure 1 shows the specific literature screening procedure.

### 3.2. Basic characteristics of the included studies

A total of 31 studies were included in this study, as shown in Table 2, which summarizes the basic characteristics of the included studies. The included studies were mainly published in 2015 and beyond, with one each in 2015 and 2017, two in 2018, four in 2019, eleven in 2020, five in 2021, four in 2022, and three relevant publications in 2023 as of February 21, 2023. Several 25 studies were conducted in mainland China (37–61) and 6 studies were conducted in Hong Kong (62–67), China. Eighteen of the studies were specifically targeted at PA, nine studies were specifically targeted at body weight status, and the other four studies examined both PA and body weight status. In terms of study design, three studies used longitudinal study design (56, 60, 61) and three studies adopted experimental study design (49, 53, 55), respectively, and the remaining 25 studies used cross-sectional study design. The sample sizes of the included studies were generally large but varied widely among studies. Ten studies had sample sizes of less than 1,000, and the remaining studies had sample sizes between 1,000 and 40,000. All studies were of Chinese adults, aged ≥18 years, and 13 of the studies were of older adults aged 60 years or older. The proportion of females in each study ranged from 46.7% to 64.3%. A variety of statistical models were used in the studies, including multi-level logistic regression, structural equation model, multiple linear regressions, generalized linear mixed models, mixed-effect difference-in-difference models, hierarchical linear model, order probit regression model, logistic regression, etc. The geographical coverage of green spaces is generally street greenery, parks, community green spaces, etc. The vast majority of studies were in urban areas, 4 studies were in urban and rural areas (42, 47, 51, 61), and only one study was in a rural area (56).

**Table 2:** Basic characteristics of the studies included in the review.

Study ID	First author (year)	Region	Study design	Sample size	Age (years)	Female (%)	Sample characteristics	Statistical model	Nonresponse rate (%)	Geographical coverage	Setting
1	Zhang, 2015	Shanghai	Cross-sectional	1,100	46–80		Adult residents	Hierarchical linear models	6	Neighborhood environment	Urban, rural
2	Zhai, 2017	Beijing	Cross-sectional	7,319	60+	63	Senior park users	Correlation analyses		Parks	Urban
3	Lu, 2018	Hong Kong	Cross-sectional	1,390	53 ± 20	51	Residents	Multi-level regression		Street greenery	Urban
4	Zhang, 2018	Guangzhou	Cross-sectional	1,003	19–59	50	Residents	Structural equation model		Greenspace	Urban
5	Wang Han, 2019	Nanning	Cross-sectional	513	18+		Adult residents	Order probit regression model	10.16	Green open space	Urban
6	Yuen, 2019	Hong Kong	Cross-sectional	554	48.1 ± 21.0	64.3	Adult residents	Pearson’s correlation analysis	0.36	Urban green space	Urban
7	Yang, 2019	Hong Kong	Cross-sectional	11,783	65+	50.5 and 53.5	Senior residents	Multi-level logistic regression		Street greenery	Urban
8	Zhang, 2019	Hong Kong	Cross-sectional	317	69.9 ± 6.8	46.7	Older adults	Negative binomial regression		Parks	Urban
9	Chen, 2020	Guangzhou	Cross-sectional	938	18–70	58	Adult residents	Multivariate analysis logistic regression	6.9	Urban green space	Urban
10	He, 2020	Wuhan	Cross-sectional	1,161	60+	53.6	Senior residents	Multi-level logistic regression	4	Street greenery	Urban
11	Huang Baishi, 2020	China	Cross-sectional	12,112	50+	53.17	Middle-aged and older residents	Multilevel structural		Neighborhood greenness	Urban, rural
12	Huang Wenzhong, 2020	Shenyang, Anshan, and Jinzhou	Cross-sectional	24,845	18–74	49	Adult residents	Two-level logistic and generalized linear mixed regression models	13.8	Community greenness	Urban
13	Lu, 2020	Shanghai	Cross-sectional	403	18–80	51.4	Adult residents	Structural equation model	5.6	Main green space in the survey area	Urban
14	Leng, 2020	Harbin	Cross-sectional	4,155	54.6 ± 10.3	47.7	Adult residents	Logistic regression		Neighborhood green space	Urban
15	Tu, 2020	Beijing	Cross-sectional	5,786	19+	53.3	Adult residents	Correlation analysis		Parks	Urban
16	Wagner, 2020	Hong Kong	Cross-sectional	306	60+	46.7	Older adults	Multiple linear regressions		Parks	Urban
17	Zhou, 2020	Guangzhou	Cross-sectional	972	60+	56.9	Older adults	Structural equation model		Neighborhood greenspaces	Urban, suburban, rural
18	Zhai, 2020	Shanghai	Cross-sectional	234	69.5 ± 7.5	43.6	Senior park users	Multiple stepwise regression analyses	8.9	Neighborhood parks	Urban
19	Zang, 2020	Hong Kong	Cross-sectional	180	65+	57	Older adults	Bivariate correlation analysis	50	Eye-Level street greenery	Urban
20	He, 2021	Wuhan	Experimental	1,020	50.8	56.9	Residents	Mixed-effect difference-in-difference models	56.2	Greenway	Urban
21	Liu, 2021	Dalian	Cross-sectional	363	60+	52.3	Older adults	Zero-inflated count models		Parks	Urban
22	Xiao, 2021	Shenyang, Anshan, and Jinzhou	Cross-sectional	24,845	45.6 ± 13.3		Adults	Generalized linear mixed models	13.8	Street view greenness	Urban
23	Xie, 2021	Wuhan	Experimental	1,020	50.8	56.6	Residents	Mixed-effects difference-in-difference regressions	56.2	Greenway	Urban
24	Yang, 2021	29 provinces/municipalities in mainland China	Cross-sectional	20,227	20.01 ± 1.74	55.48	Undergraduate students	Multilevel models		Campus green space	Urban
25	He, 2022	Wuhan	Experimental	1,020	18+		Adults	Difference-in-difference estimations and structural equation models	56.2	Greenway exposure	Urban
26	Jiang, 2022	Henan Rural	Longitudinal	39,094	55.6 ± 12.2		Adults	Generalized linear mixed models	6.3	Residential greenness	Rural
27	Xiao, 2022	Shanghai	Cross-sectional	12,780	60+	57.2	Older adults	The bootstrap-mediated analysis model comprises three models		Parks	Urban
28	Zang, 2022	Lanzhou	Cross-sectional	1773	60+		Older adults	Random forest model		Streetscape greenery	Urban
29	Cao, 2023	Guoyu	Cross-sectional	1,275	60+	45.8	Residents	Hierarchical Linear Model	17.3	Parks	Urban
30	Han, 2023	28 provinces in mainland China	Longitudinal	7,424	45+	48.2	Older adults	Cox proportional hazards models, Eneralized estimate equation	19.5	Greenness exposure	Urban
31	Zhou, 2023	23 provinces in mainland China	Longitudinal	8,318	86.5 ± 10.8	42.64	Older adults	Mixed Cox model		Residential greenness	Urban, rural

Table 3 summarizes the measures for green space, PA, and BMI in the included studies. For the measurement of green space, the majority of studies (*n* = 22) used objective assessment methods, four studies used subjective assessment methods, and five studies used a combination of subjective and objective assessment methods. The objective green space assessment methods mainly include geographical information systems (GIS), satellite remote sensing images, Baidu Street View map, Gaode Map, and Tencent Map using deep learning techniques. The assessment of subjective green space included self-reported questionnaires and field investigations. Eight studies examined the NDVI, eight studies examined the accessibility of green space, six studies examined certain characteristics of green, five studies examined the street greenness or green view index, and four studies examined the green space rate or green space perception.

**Table 3:** Measures of green space, PA, and body weight status in the studies included in the review.

Study ID	First author (year)	Type of green space measure	Detailed measure of green space	Type of PA measure	Detailed measure of PA	Type of body weight status measure	Detailed measure of body weight status
1	Zhang, 2015	Objective measure: GIS	Parkland proximity, green, and open spaces	Objective measure: pedometer	1. Total PA level 2. Total steps of walking	Objective measure	Height, weight, BMI
2	Zhai, 2017	1. Objective measure 2. On-site observations	1. Pathway length 2. Pathway design characteristic	1. Face-to-face interviews 2. On-site observations	Walking behavior		
3	Lu, 2018	1. Objective measure: GSV images, ArcGIS 2. Field observation	The quality and quantity of street greenery	Self-reported questionnaire: IPAQ	Walking, jogging, cycling		
4	Zhang, 2018	Objective measure: remote sensing images using ENVI and ArcGIS	Vegetation coverage, PA site coverage, and accessibility to the nearest greenspace	Self-reported questionnaire	The duration and frequency of PA		
5	Wang Han, 2019	1. Objective measure: Nanning city land bureau institute of green spot figure data 2. Self-reported questionnaire	Safety, accessibility, landscape quality, space environment, entertainment facilities, size of the green open space, area of the green space, and infrastructure	Self-reported questionnaire	Time and frequency of PA		
6	Yuen, 2019	Objective measure: SPOT satellite images, ArcGIS	The percentage of green space	Self-reported questionnaire: IPAQ	MET-min/week 2. PA levels		
7	Yang, 2019	Objective measure: GSV images	The level of eye-level street greenery	Self-reported questionnaire: HKTCS	Likelihood of walking 2. Walking time		
8	Zhang, 2019	Self-reported questionnaires	Park safety, attractiveness, and park feature	1. SOPARC 2. Self-reported questionnaires	The number of older adults observed being active in parks		
9	Chen, 2020	Self-report questionnaire	The distance and time from homes to green space			Self-report questionnaire	Height, weight, BMI
10	He, 2020	Objective measure: extracted from street view photographs with the machine learning technique	Street greenery index, park area, street connectivity, and land-use mix	Self-reported questionnaire: IPAQ	Duration and frequency of PA		
11	Huang Baishi, 2020	Objective measure: Landsat 5 Thematic Mapper images	Neighborhood greenness 2. NDVI	GPAQ	The intensity of work-, transport-, and leisure-related PA in a typical week; sedentary behaviors	Objective measure	Height, weight, BMI, WC, general obesity, and abdominal obesity
12	Huang Wenzhong, 2020	Objective measure: Landsat 5 Thematic Mapper satellite images, ArcGIS	NDVI, SAVI			Objective measure	Height, weight, BMI, WC, peripheral obesity, central obesity
13	Lu, 2020	Self-report questionnaire	Perceptions of green space			Self-report questionnaire	Height, weight, BMI
14	Leng, 2020	1. Objective measure: land-use data 2. First-hand field surveys	Green space ratio, green vision index, type of evergreen tree configuration, type of sports field.	Self-reported questionnaire	PA, physical inactivity	Objective measure	Height, weight, BMI
15	Tu, 2020	Objective measure	Travel distance to park and park size	Self-reported questionnaires	Park visit frequency, time, and activity type		
16	Wagner, 2020	Self-reported questionnaires	Park safety, the attractiveness of parks, PA areas, and features, park accessibility	Self-reported questionnaires	PA type, amount of PA, and intensity levels of PA in parks during a typical week.		
17	Zhou, 2020	1. Field surveys from digital photographs 2. Objective measure: Satellite-based remote sensing images	Streetscape greenery, NDVI	Self-reported questionnaire	Average time spent on PA		
18	Zhai, 2020	Objective measure	Park area, total trail length, total paved activity zone area, total natural area, presence of water, presence of outdoor fitness equipment, presence of the court	1. Objective measure: pedometer 2. Self-reported energy expenditure	1. Total steps 2. Energy expenditure 3. METs		
19	Zang, 2020	Objective measure: Baidu Street View images	Green View Index	Self-reported questionnaire: IPAQ	Walking time		
20	He, 2021	Objective measure	The East Lake greenway intervention: there are five categories representing the proximity level of residents to the greenway (0 ~ 1, 1 ~ 2, 2 ~ 3, 3 ~ 4, and 4 ~ 5 km).	Self-report	Walking behaviors		
21	Liu, 2021	Objective measure: ArcGIS combined with Baidu Map	Distance to the nearest park	A structured interviewer-administered questionnaire	Sedentary activities, utilitarian activities, leisure walking, joint leisure walking/sedentary activities, and skill-based leisure activities		
22	Xiao, 2021	Objective measure: Tencent Map, machine learning algorithms	NDVI in 800 m buffer			Objectively measured	Height, weight, BMI, WC, HC
23	Xie, 2021	Objective measure	The East Lake greenway intervention: there are five categories representing the proximity level of residents to the greenway (0 ~ 1, 1 ~ 2, 2 ~ 3, 3 ~ 4, 4 ~ 5 km).	Self-report questionnaire: IPAQ-SF12	MVPA, Total PA		
24	Yang, 2021	Objective measure: cloud-free satellite images	NDVI			Objectively measured	Height, weight, BMI, WC, WHtR
25	He, 2022	Objective measure	The East Lake greenway intervention: There are five categories representing the proximity level of residents to the greenway (0 ~ 1, 1 ~ 2, 2 ~ 3, 3 ~ 4, and 4 ~ 5 km).			Self-report questionnaire	Height, weight, BMI
26	Jiang, 2022	Objective measure: the vegetation index data was obtained from the National Aeronautics and Space Administration, MODIS.	NDVI, EVI			Objective measure	Height, weight, BMI
27	Xiao, 2022	Objective measure: ArcGIS	Density and accessibility of parks	Self-reported questionnaire	PA duration and intensity	2015 Shanghai Yangpu District Diabetes Health Records database	Height, weight, BMI
28	Zang, 2022	Objective measure: ArcGIS, Gaode Maps, Deep learning	Distance to parks, number of parks, number of street crossings, and streetscape green views	Self-reported questionnaire	Weekly hours of light PA		
29	Cao, 2023	Objective measure	1. Park accessibility 2. Density of the park	Self-reported questionnaire	Leisure walking activities: range, duration, and frequency of walking		
30	Han, 2023	Objective measure: satellite remote-sensing data	The overall greenery level: NDVI			Objective measure	Height, weight, BMI
31	Zhou, 2023	Objective measure: satellite remote-sensing data, green land cover types, and diversity	The density of green vegetation: NDVI, green land cover types, and diversity			Objective measure	Height, weight, BMI, WC

GIS, Geographic Information System; NDVI, Normalized Difference Vegetation Index; GSV, Google Street View; IPAQ, International Physical Activity Questionnaire; BMI, body mass index; MVPA, moderate-to-vigorous physical activity; MET, metabolic equivalent; PA, physical activity; SPOT, Satellite Pourl’Observation de la Terre; SOPARC, System for Observation Play and Recreation in Communities; GPAQ, Global Physical Activity Questionnaire; SAVI, Soil Adjusted Vegetation Index; EVI, enhanced vegetation index; WC, waist circumference; HC, hip circumference; WHtR, waist-to-height ratio; MODIS, Moderate Resolution Imaging Spectroradiometer; HKTCS, Hong Kong Travel Characteristics Survey.

For the measurement of PA, the majority of studies (*n* = 20) used a subjective assessment (e.g., self-reported questionnaires), only one study used an objective assessment (pedometer) (51), and one study used a combination of subjective and objective assessments (48). PA-related assessment indicators include the duration, frequency, and type of PA. For the measurement of BMI, 10 studies used an objective assessment and 3 studies used a subjective assessment (40, 44, 55). The body weight status measures included BMI, overweight, and obesity (e.g., peripheral obesity, central obesity).

### 3.3. Key findings

Table 4 summarizes the effects of green space on PA and/or BMI in adults. Since different studies have used different green space measurement or evaluation methods, direct comparisons between studies cannot be made. This study starts from the conventional indicators of green space assessment, and divides the assessment of green space into green view index, green space ratio, accessibility of green space, and the design characteristics of green space. We summarize the key findings in the following four areas.

**Table 4:** Estimated effects of green space on PA and body weight status in the studies included in the review.

Study ID	First author (year)	Estimated effects of green space		Main findings of study	
		PA	Body weight status	PA	Body weight status
1	Zhang, 2015	1. Proximity of parkland (*t* = −2.208, *p* = 0.027) and square (*t* = −3.326, *p* = 0.001) were significantly inversely associated with the likelihood of PA. 2. A 1-unit (10%) increase in the distance of parkland or square was associated with an 18% or 27% reduction in PA. 3. The green and open spaces area was not shown to be significantly associated with PA (Coefficient = 0.093, *p* = 0.407).	1. A 1-SD increase in the proximity of parkland (*t* = 2.238, *p* = 0.026) was associated with a 10% increase in BMI. 2. A 1-unit (10%) decrease in the proximity of parkland (*t* = 3.308, *p* = 0.002) will increase by 18% in overweight/obesity. 3. A 1-unit (10%) increase in green and open space areas (*t* = −0.118, *p* = 0.008) was accompanied by a 12% BMI reduction.	1. Parkland and square proximity related to the likelihood PA: − 2. The distance of parkland or square related to the PA: − 3. Green and open space area in the 500 m buffer related to walking: 0	1. Parkland proximity related to BMI: + 2. Parkland proximity related to overweight/obesity: − 3. Green and open space areas related to BMI: −
2	Zhai, 2017	1. At Rendinghu Park and Yuetan Park, pathway length is correlated favorably with the number of seniors observed [*r* (32) = 0.58, *p*<0.01 and *r* (39) = 0.52, *p*<0.01, respectively]. 2. Seniors in Rendinghu Park utilize flower-adorned and step-free pathways more frequently (*p*<0.001) and, respectively. 3. In Yuetan Park, paths without connections to activity zones are used more frequently than those that connect with two activity zones (*p*<0.001). 4. In neither of the parks, there is a correlation between the number of seniors observed and the type of pathway, the amount of shade, the degree of enclosure, the existence of water on the side, or the visual proximity to water.		1. Park pathway length related to seniors walking: + 2. Soft or even pavement, seats, flowers, and lighting fixtures are preferred by seniors when designing walkways. 3. Pathways that are long, between 3 and 3.9 m wide, and disconnected from activity zones are attractive to seniors. 4. Additional design elements of the walkway, such as its proximity to water, the presence of shade, the availability of lateral visibility and a visual connection to the water, and the absence of a visual connection to prominent landmarks, may also promote senior walking.	
3	Lu, 2018	1. Compared to participants exposed to low amounts of street greenery, those exposed to large amounts had a significantly higher likelihood of regularly engaging in recreational green PA (OR = 1.20, 95%CI = 1.08, 1.33). 2. Compared to residents who were exposed to low-quality street greenery, residents who were exposed to high-quality street greenery were more likely to engage in frequent recreational green PA (OR = 1.10, 95%CI = 1.05, 1.25). 3. Compared to participants exposed to low levels of total park space in the buffer, those exposed to high levels of total park area had a higher chance of developing PA (OR = 1.22, 95%CI = 1.10, 1.36).		1. High quality and quantity of street greenery related to recreational PA: + 2. Medium quality and quantity of street greenery related to recreational PA: 0 3. High total park area related to recreational PA: + 4. Medium total park area related to recreational PA: 0	
4	Zhang, 2018	Exposure to green spaces significantly improves PA (path coefficient = 0.14, C.R. = 3.213, *p* <0.01).		Green space exposure related to the PA level: +	
5	Wang Han, 2019	1. There is a strong positive correlation between accessibility and residents’ PA. 2. There is no evident link between inhabitants’ PA and the environment of natural spaces, the quality of the landscape, or safety. 3. There is a substantial correlation between infrastructure (*r* = 0.220, *p*<0.01), green space area (*r* = 0.0003998, *p*<0.1), open space size (*r* = 0.000107, *p*<0.1), and entertainment facilities.		1. Accessibility related to PA: + 2. Nature space environment, landscape quality, and safety related to PA: 0 3. Infrastructures related to PA: + 4. The area of green space related to PA: + 5. The size of open space related to PA: +	
6	Yuen, 2019	1. MET-min/week had a statistically significant relationship (*r* = 0.092; *p*<0.05) with the proportion of green space. 2. In terms of IPAQ levels, the subgroups with “medium” and “high” green space tended to perform moderate-to-high levels of PA, whereas those with poor green space mostly performed at a moderate level.		1. Green space percentage related to MET-minutes/week: + 2. Green space level related to IPAQ level: +	
7	Yang, 2019	1. The likelihood of walking was significantly correlated with the presence of street greenery (OR = 1.206, 95%CI = 1.039, 1.400). 2. Street greenery was positively correlated with overall walking time (OR = 0.187, 95%CI = 0.071, 0.304); walking time for older adults increased by almost 0.2 standard deviations for every unit increase in street greenery.		1. Street greenery related to the odds of walking: + 2. Street greenery related to walking time: +	
8	Zhang, 2019	1. The number of active older adults in Hong Kong parks was positively correlated with the types of activity space, Wald X^2^ (6) = 538.18, *p* <0.001. 2. Among older adults in Hong Kong parks, perceived park distance (*β* = 0.05, *p* = 0.38), attractiveness (*β* = 0.10, *p* = 0.09), characteristics (*β* = 0.01, *p* = 0.94), and safety (*β* = 0.10, *p* = 0.11) did not significantly correlate with park-based PA.		1. The types of activity areas related to the number of active older adults in parks: + 2. Perceived park safety, attractiveness, park features, and park distance related to park-based PA: 0	
9	Chen, 2020		When an urban green space was within a kilometer, it was linked to a lower risk of being overweight or obese (*β* = 0.320, *p* <0.01).		Accessibility of green space related to obesity: −
10	He, 2020	1. There was a significant correlation between street greenery and the likelihood of getting 300 min or more of PA per week (OR = 1.287, 95%CI = 1.105, 1.498, *p* < 0.001). 2. Neither the frequency nor the duration of PA was significantly correlated with park area.		1. Street greenery related to the odds of PA: + 2. Park area related to the frequency of PA: 0 3. Park area related to the total time of PA: 0	
11	Huang Baishi, 2020	NDVI did not significantly correlate with the amount of time spent sedentary (*β* = 0.62, 95% CI −31.20 to 29.96) or transportation- and leisure-related PA (*β* = 47.06, 95% CI −33.05 to 127.16).	1. There is a substantial negative correlation (odds = 0.73, 95% CI = 0.58, 0.92) between residential greenness and overweight/obesity. 2. There is a substantial negative correlation (odds = 0.55, 95% CI = 0.33, 0.91) between the level of residential greenness and abdominal obesity.	Green space related to transportation and leisure PA time and sedentary behavior: 0	Residential greenness was related to overweight/obesity: − 2. Residential greenness related to abdominal obesity: −
12	Huang Wenzhong, 2020		A 0.18 kg/m^2^ (95% CI = 0.24, 0.11) lower BMI, 20% (95% CI = 26, 13%) lower odds for peripheral obesity, and 12% (95% CI = 17, 7%) lower odds for central obesity were all linked with each interquartile range (0.17 unit) rise in NDVI500-m.		Community greenness was related to BMI: − Community greenness was related to the odds for peripheral obesity: − Community greenness was related to odds for central obesity: −
13	Lu, 2020		1. The perception of large parks positively correlates to BMI (*β* = 0.169, SE = 0.087, *p* = 0.053). 2. The perception of small parks has a negative association with BMI (*β* = −0.174, SE = 0.100, *p* = 0.082).		Perception of large parks related to BMI: + 2. Perception of small parks related to BMI: −
14	Leng, 2020	1. Compared to neighborhoods with a Green Space Ratio of more than 28%, people in neighborhoods with a Green Space Ratio lower than 28% have a higher risk of being physically inactive (OR = 0.62, 95%CI = 0.44, 0.87, *p* = 0.006). 2. Participants with a decreased risk of inactivity were those who resided in areas with a Green View Index of more than 15% (OR = 0.53; 95% CI = 0.39, 0.72, *p* = 0.000). 3. Physical inactivity and the evergreen tree configuration type were not significantly correlated. 4. The type of sports field and inactivity were not significantly correlated.	1. Residents had an increased risk of being overweight or obese in areas where the proportion of green space was lower than 28% (OR = 1.22, 95% CI = 1.01, 1.46) 2. Residents had an increased probability of being overweight or obese in areas with a green view index below 15% (OR = 1.28, 95% CI = 1.09, 1.52). 3. The presence of evergreen trees was linked to overweight/obesity (OR = 1.44, 95% CI = 1.09, 1.91).	1. Green space ratio lower than 28% related to physical inactivity: + 2. Green view index of more than 15% related to physical inactivity: − 3. Evergreen tree configuration type related to physical inactivity: 0 4. Sports field type related to physical inactivity: 0	1. Green space ratio equal to or lower than 28% related to overweight/obesity: + 2. Green view index equal to or lower than 15% related to overweight/obesity: + 3. Evergreen trees configuration related to overweight/obese: +
15	Tu, 2020	1. The ratio of visitors and travel distance had a negative correlation (*r* = −0.344, *p*<0.001). 2. There was no evident correlation between park size and visitation ratio.		1. Travel distance related to the ratio of visitors: − 2. Park size related to the ratio of visitors: 0	
16	Wagner, 2020	1. There was no correlation between perceived park features and energy use [*β* = 0.05, *t* (253) = 0.77, *p* = 0.44]. 2. There was no correlation between energy use and perceived park time distance [*β* = 0.05, *t* (253) = 0.83, *p* = 0.41].		1. Perceived park features related to energy expenditure: 0 2. Perceived park time distance and energy expenditure: 0	
17	Zhou, 2020	The amount of greenery in the neighborhood streetscape was correlated with older persons’ average time spent participating in PA (Standardized estimates = 0.18, *p*<0.01).		Neighborhood streetscape greenness related to PA time: +	
18	Zhai, 2020	1. The number of steps taken was positively correlated with the park’s overall natural area (*β* = 0.158, *p* = 0.015), as well as with the existence of outdoor exercise equipment (*β* = 0.149, *p* = 0.021). 2. The presence of outdoor exercise equipment was significantly correlated with seniors’ energy expenditure (*β* = 0.161, *p* = 0.024).		1. Total natural area in the park related to total steps: + 2. The presence of outdoor fitness equipment related to total step: + 3. The presence of outdoor fitness equipment related to energy expenditure: +	
19	Zang, 2020	The older adult’s walking duration is significantly impacted by the green view index (*β* = 0.137, *p* = 0.05).		Green view index related to walking time: +	
20	He, 2021	1. The greenway intervention significantly increased the amount of time people walked each week. 2. The walking benefits conferred by the greenway can be extended to residents living within 2 km of the greenway access.		The greenway intervention related to walking time, especially for residents living within two kilometers of the greenway: +	
21	Liu, 2021	Distances of less than 800 m to the nearest park have a negative impact on how often older adults participate in sedentary activities, and distances of 800–1,200 m may support those who regularly engage in recreational walking.		800–1,200 m park distance related to leisure walking: +	
22	Xiao, 2021		Each interquartile range (IQR = 3.6%) increase in street view greenness was associated with a 0.15 kg/m^2^ (95% CI: −0.22, −0.09) decrease in BMI and 0.23 cm (95% CI: −0.35, −0.11) reduction in HC, and was associated with 7% lower odds of overweight (OR = 0.93, 95% CI: 0.90, 0.96) and 18% lower odds of obesity (OR = 0.82, 95% CI: 0.76, 0.89).		Community-level street view greenness related to body weight: −
23	Xie, 2021	1. In the follow-up period, the MVPA and overall PA of the exposed group significantly increased by 62.7 min/week and 448.9 MET-min/week in absolute terms and by 9.5% and 10.4% in percentage terms, respectively, compared with the baseline level. 2. The effect of the greenway intervention on MVPA decreased by 0.046 SD as the distance increased by one SD. The same result was held for overall physical activity. The effect of the greenway intervention decreased with distance, with an effect size of 0.061.		1. The Greenway intervention related to MVPA and total PA: + 2. PA benefits of greenway interventions decrease with distance.	
24	Yang, 2021		NDVI in and around the campus environment was negatively associated with the odds of obesity to varying degrees.		Campus green space related to obesity: −
25	He, 2022		1. The results showed that the treatment group experienced a minor BMI reduction, while the BMI increased in the control group. 2. Both men and women in the treatment group experienced a minor BMI reduction after the intervention.		Greenway interventions related to BMI: −
26	Jiang, 2022		An IQR increase in EVI500-m was linked with reduced odds of obesity (OR = 0.77, 95%CI: 0.72–0.82) and BMI level (*β* = −0.41 kg/m^2^, 95%CI: − 0.48 to −0.33 kg/m^2^)		Higher residential greenness was correlated with lower odds of obesity and BMI level:−
27	Xiao, 2022	The density and accessibility aspects of parks can be a great stimulus to PA for older adults.	The density and accessibility of parks can reduce BMI in older adults.	The density and accessibility of parks are related to PA for older adults: +	The density and accessibility of parks are related to the BMI of older adults: −
28	Zang, 2022	1. Streetscape greenery has the most significant impact on older adults’ light PA. 2. A greater distance to the park within 1 km is associated with a longer time spent on light PA.		Both streetscape greenery and distance to parks within one kilometer were associated with light PA: +	
29	Cao, 2023	1. The higher the parking density (0.850) and the better the road lighting at night (0.333), the more likely the older adults were to spend >150 min per week on leisure walking. 2. The park density (1.996) showed a significant positive effect on older adult people’s participation in recreational walking activities when their recreational walking space was expanded to 800–1,500 m, and the significance level and degree of effect were significantly higher.		Park density is associated with older adults’ participation in leisure walking activities: +	
30	Han, 2023		The study found that the higher the exposure to greenness, the less likely people were to be overweight or obese (HR = 0.806, 95% CI = 0.754 ~ 0.862).		Greenness exposure is associated with the risk of being overweight or obese: −
31	Zhou, 2023		Single exposure models showed that higher levels of residential greenness, tree coverage, and tree-to-shrub/grass ratios were negatively associated with peripheral overweight/obesity and central obesity. Increases in shrubland, grass, and green diversity were associated with lower odds of peripheral overweight/obesity.		Residential greenness and green diversity are associated with body weight status: −

PA, physical activity; BMI, body mass index; IPAQ, International Physical Activity Questionnaire; MET, metabolic equivalent; NDVI, Normalized Difference Vegetation Index; MVPA, moderate-to-vigorous physical activity; IQR, interquartile range; EVI, enhanced vegetation index; correlation: + positively; − negatively; 0 insignificantly.

First, all studies that used street-level green view index as a measure of green space showed that street-level green view index was significantly positively associated with PA and significantly negatively associated with BMI among Chinese adults. The green view index refers to the proportion of green scenes such as natural landscapes and vegetation in people’s view (68). Street greenery, street greenness, and so on in the article represent the street-level green view index. For example, Studies have shown a positive relationship between street greenery and total walking time, with each standard deviation increase in street greenery being associated with an increase of approximately 0.2 standard deviations in walking time for older adults (64). Lu found that participants exposed to large amounts of street greenery were significantly more likely to engage in regular recreational greening activities compared to participants exposed to low amounts of street greenery (62). Moreover, research has revealed that residents’ odds of being overweight or obese are higher in areas with a green view index of less than 15% (45). And with every interquartile range increase in street view greenness, BMI decreases by 0.15 kg/m^2^, the odds of being overweight decrease by 7%, and the odds of being obese decreased by 18% (52). Furthermore, in a natural experimental study, a greenway intervention was found to have a significant positive effect on walking time, especially for residents living within two kilometers of a greenway (49). Another recent study showed that greenway interventions were effective in reducing weight (55). However, in general, both longitudinal and experimental studies on the effects of green view index on PA and body weight status are scarce, and further research is needed in the future.

Second, most studies showed that green space ratio were associated with PA and/or weight status among Chinese adults, but some studies also showed no correlation. Green space ratio is the ratio of total green space to neighborhood area. The NDVI, green space rate, and so on in the article all represent the green space ratio. For example, compared to neighborhoods with a green space ratio of more than 28%, persons in neighborhoods with a green space ratio lower than 28% are more likely to be physically inactive (45). Wang et al. concluded that urban green spaces and open spaces play an important role in promoting PA among residents, especially women and the older adults, and their study found that the area of green space was significantly associated with residents’ PA (39). However, the Huang et al. study found no connection between the NDVI and time spent on sedentary activities, leisure-related PA, or time spent traveling (42). The author explains that the research may focus on middle-aged and older adult people, who typically spend less time on traveling and leisure-related PA. NDVI may be difficult to affect the traveling and leisure-related PA of the older adults. In addition, half of the participants live in rural areas where the vegetation is used for agricultural purposes and is not suitable for participating in sports activities related to transportation and leisure. It can be seen that individual differences, regional differences, and PA types of the research subjects may have different impacts on the results. Furthermore, in the reviewed studies, there was a significant negative correlation between the green space rate and obesity among Chinese adults. For example, in a longitudinal study design, scholars found that higher rates of green space reduced the risk of being overweight or obese (60).

Third, the studies reviewed found a significant relationship between the accessibility of green space and PA and/or obesity among Chinese adults. The accessibility of green space in this study mainly refers to the convenience of reaching the green space from the residence, and the indicators for evaluation are mainly distance and time, etc. A previous study showed that the distance to a park was significantly and inversely related to the likelihood of residents to participate in PA. For every 1-unit (10%) increase in distance to a park, residents’ participation in PA decreased by 18% or 27% (69). Wang et al. showed that the accessibility of green space was significantly and positively correlated with the PA of residents (39). A research study found that maximum park visits declined exponentially as travel distance to the park increased (46). Studies of older adults have found that being less than 800 m from the nearest park has a negative impact on sedentary activities, while a distance of 800–1,200 m may support those who regularly participate in recreational walking (50). In addition, easier access to urban green space was associated with lower odds of being overweight or obese when it was within 1 km of an urban green space (40). And a study by Xiao et al. reported that park accessibility was significantly and negatively associated with BMI in older adults (57). The accessibility of green spaces can reflect the ease of residents to reach green spaces such as parks, and also reflects the level of green space service capacity from the side. Based on the results of previous studies, it is recommended to improve the accessibility of green spaces around residences to promote residents’ PA and reduce the risk of overweight and obesity.

Fourth, design characteristics of green space had an effect on PA and/or obesity among Chinese adults, but there were some studies showing no statistically significant results. The design characteristics of green spaces in this study mainly included variables such as outdoor fitness equipment in green spaces, the configuration of evergreen trees, the diversity of greenery, and residents’ perceptions of green spaces. A study from Hong Kong showed that older adults’ perceptions of park safety, attractiveness, and park characteristics were not significantly related to park-based exercise in Hong Kong’s parks (65). And some scholars have also shown that space environment and landscape quality are not significantly correlated with residents’ PA (39). However, studies have also shown that the total number of steps engaged by older adults is positively correlated with the presence of outdoor fitness equipment. And the energy expenditure of older adults was positively correlated with the presence of outdoor fitness equipment (48). Zhai and Baran showed that parkway length was positively associated with walking among older adults and that soft or level pavement, seating, flowers, and lighting fixtures were preferred when designing sidewalks (37). In addition, the research by Leng et al. shows that the design characteristics of green spaces (such as the evergreen tree configuration type in green spaces) are related to the overweight/obesity of residents (45). One longitudinal study showed that levels of residential greenery, tree cover, and tree-to-shrub/grass ratios were negatively associated with peripheral overweight/obesity and central obesity. Increased shrub, grass, and green diversity were associated with lower odds of peripheral overweight/obesity (61). The design characteristics of green spaces affect the perception and attractiveness of green spaces to residents. It is recommended that outdoor fitness equipment, increased diversity of greenery, and safety of green spaces be configured to increase the opportunities for residents to participate in PA.

### 3.4. Study quality assessment

Table 5 lists the study quality assessment’s global and criterion-specific scores. The average score for the included studies was 7 (ranging from 5 to 11). The research questions and objectives of all the studies included in the review were clearly stated, the study population was identified and defined with a 50% participation rate, participants were drawn from the same or related populations during the same period, and inclusion and exclusion criteria were pre-established and uniformly applied to all potential participants. Twelve studies investigated various exposure levels about the outcome. Twenty five studies used valid and reliable exposure measures. Twenty two studies implemented valid and reliable outcome measures. Twenty four studies measured and statistically adjusted for key potential confounding variables between exposure and outcome. In addition, six studies had fairly long follow-up periods, sufficient to observe changes in outcomes. And three studies assessed exposure more than once during the study period, and two studies had less than 20% loss to follow-up after baseline. In contrast, none of the studies provided a rationale for sample size using power analysis, none measured relevant exposures before outcomes, and none had outcome assessors blinded to the exposure status of participants.

**Table 5:** Study quality assessment.

Criterion	Study ID																														
	1	2	3	4	5	6	7	8	9	10	11	12	13	14	15	16	17	18	19	20	21	22	23	24	25	26	27	28	29	30	31
1. Was the research question or objective in this paper clearly stated?	1	1	1	1	1	1	1	1	1	1	1	1	1	1	1	1	1	1	1	1	1	1	1	1	1	1	1	1	1	1	1
2. Was the study population clearly specified and defined?	1	1	1	1	1	1	1	1	1	1	1	1	1	1	1	1	1	1	1	1	1	1	1	1	1	1	1	1	1	1	1
3. Was the participation rate of eligible persons at least 50%?	1	1	1	1	1	1	1	1	1	1	1	1	1	1	1	1	1	1	1	1	1	1	1	1	1	1	1	1	1	1	1
4. Were all the subjects selected or recruited from the same or similar populations (including the same time period)? Were inclusion and exclusion criteria for being in the study pre-specified and applied uniformly to all participants?	1	1	1	1	1	1	1	1	1	1	1	1	1	1	1	1	1	1	1	1	1	1	1	1	1	1	1	1	1	1	1
5. Was a sample size justification, power description, or variance and effect estimates provided?	0	0	0	0	0	0	0	0	0	0	0	0	0	0	0	0	0	0	0	0	0	0	0	0	0	0	0	0	0	0	0
6. For the analyses in this paper, were the exposure(s) of interest measured prior to the outcome(s) being measured?	0	0	0	0	0	0	0	0	0	0	0	0	0	0	0	0	0	0	0	0	0	0	0	0	0	0	0	0	0	0	0
7. Was the timeframe sufficient so that one could reasonably expect to see an association between exposure and outcome if it existed?	0	0	0	0	0	0	0	0	0	0	0	0	0	0	0	0	0	0	0	1	0	0	1	0	1	1	0	0	0	1	1
8. For exposures that can vary in amount or level, did the study examine different levels of the exposure as related to the outcome (e.g., categories of exposure or exposure measured as continuous variable)?	0	0	1	0	0	1	0	0	1	0	0	1	0	1	1	0	0	0	0	1	0	0	1	0	1	1	0	1	0	0	1
9. Were the exposure measures (independent variables) clearly defined, valid, reliable, and implemented consistently across all study participants?	1	1	1	1	1	1	1	0	0	1	1	1	0	1	1	0	1	1	1	1	1	1	1	1	1	1	0	1	0	1	1
10. Was the exposure(s) assessed more than once over time?	0	0	0	0	0	0	0	0	0	0	0	0	0	0	0	0	0	0	0	0	0	0	0	0	0	1	0	0	0	1	1
11. Were the outcome measures (dependent variables) clearly defined, valid, reliable, and implemented consistently across all study participants?	1	0	1	0	0	1	1	1	0	1	1	1	0	0	0	0	0	1	1	1	1	1	1	1	1	1	1	1	1	1	1
12. Were the outcome assessors blinded to the exposure status of participants?	0	0	0	0	0	0	0	0	0	0	0	0	0	0	0	0	0	0	0	0	0	0	0	0	0	0	0	0	0	0	0
13. Was loss to follow-up after baseline 20% or less?	0	0	0	0	0	0	0	0	0	0	0	0	0	0	0	0	0	0	0	0	0	0	0	0	0	1	0	0	0	1	0
14. Were key potential confounding variables measured and adjusted statistically for their impact on the relationship between exposure(s) and outcome(s)?	1	0	1	1	1	0	1	1	0	1	1	1	0	1	0	1	1	1	0	1	1	1	1	1	1	1	1	0	1	1	1
Total score	7	5	8	6	6	7	7	6	5	7	7	8	5	7	6	5	6	7	6	9	7	7	9	7	9	11	6	7	6	10	10

## 4. Discussion

This study systematically reviews the scientific evidence on the effects of green space on PA and body weight status in Chinese adults. A total of 31 studies met the inclusion criteria for this study, including 25 cross-sectional studies, 3 longitudinal studies, and 3 experimental studies. Although green space was found to be significantly positively correlated with PA and negatively correlated with BMI, overweight, and obesity among Chinese residents in most studies, some studies showed no correlation. It is still difficult to draw a clear conclusion about the relationship between green space and PA and the body weight status of residents.

In this study, we found that street-level green view index were positively associated with residents’ PA and negatively associated with BMI in studies focusing on street green space (52, 58, 64). Green space ratio in local spaces was positively correlated with PA for most of the studies reviewed, but negatively correlated with BMI for all studies (60). In studies focusing on the accessibility of green space, most studies showed that PA and body weight status of Chinese adults were significantly correlated with park accessibility (39, 40). In addition, since the design of green spaces contains several dimensions, such as the diversity of green plants and the configuration of sports facilities in green spaces. Therefore different design characteristics may have different effects on the physical activities of residents. For example, one study showed that natural space environment, landscape quality, and safety were not significantly associated with residents’ PA (65). But one study reported that the presence of outdoor fitness equipment in parks was positively correlated with PA among older adults (48). There are relatively few studies on green space characteristics and weight status of Chinese adults, which can be further explored in the future. In addition, a total of three studies in this systematic review used an experimental design, and all showed positive effects of green space interventions on residents’ PA and weight status (49, 53, 55), but there are still relatively few studies using an experimental design, and further relevant studies are needed to support this result in the future.

In general, the main findings of the systematic review are similar to those of studies in a number of developed and developing countries. For example, in developed countries, a previous study explored the relationship between accelerometer-based measures of PA levels of older adults in Spain and their visits to green spaces. It showed that visiting green spaces was positively related to older adults’ PA levels (70). And in a study of Japanese older adults, the presence of parks or green spaces was found to be positively correlated with the frequency of PA (71). Another study from the Netherlands showed that the odds of children being overweight decreased with increasing exposure to green space in a 3,000 m buffer zone (72). Moreover, among developing countries, a study from Turkey showed that short distances to urban green spaces, many trees, exercise equipment and picnic areas were positively associated with the frequency of physical activity of residents (73). And in one Dutch city, the amount of green and recreational space, especially parks and sports fields, within 300 and 500 m of participants’ homes was positively correlated with time spent cycling (74). Studies of Mexican adults showed that exposure to green space was associated with lower BMI, even after adjusting for important confounders (75). In our systematic review, most studies also showed that green space had a positive effect on PA and weight status of Chinese residents. This further confirms that environmental interventions can be used to promote healthy behaviors and increase physical activity levels among residents.

However, contrary findings also exist, for example, a study from New Zealand showed no significant correlation between neighborhood green space and overweight and obesity in children (76). In addition, a cross-sectional study of 3,679 Scottish adults showed no significant correlation between the availability of green space in the community and the total PA of residents (77). There were also studies in the literature included in this study that did not find a relationship between green space and PA or weight status. The potential explanation for this inconsistency between studies could be due to differences in the way green space was measured or the indicators were chosen to assess it between studies. The green space data sources of the included studies differed, such as from satellite remote sensing images, self-reported questionnaires, individual street maps, and regional government databases, and thus would provide varying degrees of accuracy. For example, studies have shown that street-level green views assessed using Google Street View images show a significant correlation with the odds of residents bicycling, while overhead green views assessed by NDVI are not correlated with the odds of bicycling (78). In addition, confounding may be a potential factor for differences in results between the studies included in this systematic review. Most studies included only socio-demographic variables for adjustment, such as gender, age, education level, and economic level, and few studies included variables such as social cohesion, air quality, and noise for adjustment.

This study systematically and comprehensively reviewed the existing literature on the effects of green space on PA and body weight status of Chinese adults. It is recommended that future studies should research environmental effects on human health behaviors for different green space characteristics, exposure doses, and different populations to better understand the relationship between the environment and human health. Second, most of the current studies mainly use cross-sectional study designs, which cannot explain the causal relationship between green space and health, and more longitudinal studies or experimental studies need to be used in the future to further explore the effects of green space on residents’ health. Third, the dose and duration of green space exposure affecting PA and weight status of Chinese residents are still not fully understood, and the dose–response of green space exposure and the duration of its effects on residents’ health needs to be analyzed in the future. Finally, further analysis of the mediating factors affecting the relationship between green space and residents’ health, such as socio-environmental factors, air quality, and noise, is needed in the future, and these complex causal relationships between environmental characteristics need to be further elucidated in future studies.

The results of the systematic review provide some guiding information for the development of relevant sectoral policies and environmental intervention programs. However, there was a large heterogeneity of green space exposure and study participants in the included studies; and there was a large variation in the assessment of green space and PA across studies. For example, for the assessment of green space, some studies assessed the scale of green space while others assessed the perception of green space; for the assessment of PA, accelerometry and self-reported questionnaires were included. In addition, different studies control for different confounding factors, which can produce some heterogeneity. The above reasons prevented us from conducting a quantitative and comprehensive analysis. Second, not all of the included studies used objective assessments of green space and PA measures, and some of the studies used self-report questionnaires, so the questionnaire results may have produced some recall biases. Third, only published literature in English was included in this study, excluding literature in other languages, and there may be some selection biases.

## 5. Conclusion

This systematic review provides a comprehensive overview of the effects of green space on PA and body weight status among Chinese adults. Green view index, green space accessibility, and green space ratio were found to be positively associated with PA and negatively associated with BMI. Furthermore, green space interventions were effective in increasing PA levels and managing healthy weight among Chinese adults. However, the relationship between the design characteristics of green space and PA and weight status still needs to be further explored. It is recommended that more longitudinal and quasi-experimental studies be conducted in the future to explore the effects of green space on PA and the body weight status of residents and the causal relationships among them, to provide some evidence to support relevant policy formulation and intervention program design.

## Author contributions

YS: study concept and design and drafting of the manuscript. HL: data extraction and analysis. HY: study supervision. All authors contributed to the article and approved the submitted version.

## Funding

This study was supported by the National Social Science Foundation of China (17CTY020, 20BTY004), Beijing Social Science Foundation of China (21YTA009), and the Tsinghua University “Shuang Gao” Scientific Research Program (2021TSG08208), the Tsinghua Education Reform Project (2021ZY01_01), the Tsinghua Graduated Education Reform Project (202303J039).

## Conflict of interest

The authors declare that the research was conducted in the absence of any commercial or financial relationships that could be construed as a potential conflict of interest.

## Publisher’s note

All claims expressed in this article are solely those of the authors and do not necessarily represent those of their affiliated organizations, or those of the publisher, the editors and the reviewers. Any product that may be evaluated in this article, or claim that may be made by its manufacturer, is not guaranteed or endorsed by the publisher.
